# Radiologic analysis of CT imaging patterns and clinical correlations in hospitalized pediatric COVID-19 patients

**DOI:** 10.3389/fradi.2025.1571672

**Published:** 2025-04-24

**Authors:** Mehrnoosh Aghabeygiha, Seyed Alireza Fahimzad, Shima Behzad, Rasoul Hossein Zadeh, Farzad Sheikhzadeh, Yasaman Tamaddon, Mahmoud Hajipour, Reza Hossein Zadeh, Ali Neyriz, Neda Pak, Armin Shirvani, Amirhossein Hosseini, Mitra Khalili

**Affiliations:** ^1^ School of Medicine, Shahid Beheshti University of Medical Sciences, Tehran, Iran; ^2^ School of Medicine, Islamic Azad University of Medical Sciences, Tehran, Iran; ^3^ Faculty of Medicine, Mashhad University of Medical Sciences, Mashhad, Iran; ^4^ School of Medicine, Iran University of Medical Sciences, Tehran, Iran; ^5^ Faculty of Medicine, Urmia University of Medical Sciences, Urmia, Iran; ^6^ Pediatric Gastroenterology, Hepatology and Nutrition Research Center, Research Institute for Children’s Health, Shahid Beheshti University of Medical Sciences, Tehran, Iran; ^7^ Student Research Committee, Faculty of Medicine, Mashhad University of Medical Sciences, Mashhad, Iran; ^8^ Radiology Department, Faculty of Medicine, University of Medical Sciences, Qazvin, Iran; ^9^ Associate Professor of Radiology, Children’s Medical Center, Tehran University of Medical Sciences, Tehran, Iran; ^10^ Radiology Department, Mofid Children’s Hospital, Shahid Beheshti University of Medical Sciences, Tehran, Iran

**Keywords:** COVID-19, CT scan, infection, ICU—intensive care unit, pediatric

## Abstract

**Background and objective:**

COVID-19 has emerged as a global pandemic affecting individuals of all ages. The disease can lead to severe complications and even death, particularly due to pulmonary involvement. Contrary to popular belief, children can also experience significant complications from COVID-19. To date, there have been limited studies focusing on pulmonary manifestations in pediatric patients with COVID-19. This study aims to investigate the imaging patterns (CT scans) in children diagnosed with COVID-19 in Iran.

**Materials and methods:**

This retrospective study analyzed data from hospitalized children with COVID-19 in Tehran from March 2020 to September 2020. Information collected included demographic details (sex and age), previous medical history, clinical manifestations, vital signs at admission, laboratory findings, and imaging results, including CT scan and chest x-ray.

**Results:**

252 patients were included, with a mean age of 71.2 ± 59.42 months; 58.3% were male. Fever was the most prevalent symptom, occurring in 67.4% of cases. The most common underlying condition was oncological disorders, present in 85% of patients. Notably, 52% required admission to the ICU, and 1.8% needed intubation. CT scans revealed that the most frequent lung involvement patterns were mixed patterns and consolidation, with bilateral involvement being the most common. The mean CT score was calculated at 3 ± 4. Abnormal CT findings were associated with a poorer prognosis, and correlations were observed between specific CT findings and clinical manifestations.

**Conclusion:**

Chest CT manifestations offer valuable insights for assessing pediatric patients with COVID-19, especially in severe cases and those with pre-existing health conditions. Integrating clinical evaluations with radiological scoring systems facilitates early identification of disease severity.

## Introduction

In January 2020, severe acute respiratory syndrome coronavirus 2 (SARS-CoV-2) was identified as the cause of pneumonia cases first reported in Wuhan, China. Soon after, the virus started spreading around the world (1). By March 2020, the World Health Organization declared COVID-19 a pandemic (2). While COVID-19 primarily affects adults, there has been a notable increase in reported cases among children (3). Current data indicates that children generally experience a lower incidence of symptomatic disease and tend to have milder illness (4, 5). Common symptoms in pediatric cases include fever and cough, which are also prevalent in other childhood viral infections (6).

In the United States, the hospitalization rate for children under 18 stands at 8 per 100,000, significantly lower than the 164.5 per 100,000 rate observed in adults (7). This disparity may be attributed to distinct immune responses in children, including elevated levels of innate IgM antibodies and rapid natural antibody production, as highlighted by Carsetti et al. (8). Factors such as variations in T cell responses in adults, differing ACE-2 expression levels in children, and the presence of other respiratory viruses also contribute to this phenomenon (9). Additionally, children typically have fewer underlying health conditions and exhibit better lung regeneration capabilities than adults (10).

Nucleic acid testing using reverse transcriptase-polymerase chain reaction (RT-PCR) remains the gold standard for diagnosing COVID-19. However, delays in result turnaround can occur due to limitations in test kit availability, laboratory capacity, and trained personnel (11). Moreover, test sensitivity can be suboptimal, with detection rates ranging between 50% and 72% for nasal swab samples (12, 13). Consequently, the role of chest imaging in diagnosing COVID-19 has gained attention.

Research indicates that computed tomography (CT) chest imaging may offer higher sensitivity and prognostic value than traditional chest x-rays (14). Some studies have found that patients with normal chest radiographs often exhibit subtle abnormalities on CT scans (15). In fact, individuals who initially test negative via RT-PCR but show abnormal findings on chest CT may later test positive during their hospital stay (16). This suggests that CT imaging could be crucial for early detection and timely isolation of COVID-19 cases.

However, there is limited evidence regarding how COVID-19 manifests in pediatric imaging findings. A systematic review identified ground-glass opacities (GGO) and consolidations or pneumonic infiltrates as the most common abnormalities in pediatric COVID-19 cases (17). Remarkably, over one-third of affected children had normal chest CT scans. Furthermore, data on the relationship between radiological findings indicative of COVID-19 severity and clinical presentation in children remains sparse. A retrospective study by Ali et al. (18) found significant associations between severe-to-critical illness and abnormal radiological findings.

Therefore, this study aims to reveal the clinical characteristics and imaging features of COVID-19 in children. The goal is to establish standardized clinical and imaging approaches that accurately predict disease severity in children.

## Materials and methods

### Study design and population

This study was a cross-sectional investigation conducted among children under 18 years of age who were confirmed cases of COVID-19 based on clinical findings and RT-PCR test results. The study population comprised patients admitted to the Mofid Children's hospital in Tehran, Iran, from March 2020 to September 2020. Patients with incomplete medical records, a history of respiratory or allergic diseases, recent respiratory infections, and congenital heart and lung disorders were excluded from the study. The minimum required sample size was calculated to be 86 children based on previous findings indicating abnormal pulmonary manifestations in 66% of children with COVID-19 (19).

### Data collection

Data were collected through a retrospective review of patient records, which included information on patients' backgrounds, clinical manifestations, laboratory findings, and disease outcomes during hospitalization. Vital signs at admission and laboratory findings (e.g., blood cell counts, inflammatory markers, coagulation tests) were documented. The severity of the disease was determined based on the Iranian national guidelines for the diagnosis and management of COVID-19 in pediatric patients (1).

### Inclusion and exclusion criteria

Patients with symptoms suggestive of infection but without respiratory distress or abnormal imaging findings are classified as having mild disease. Moderate disease is defined by imaging showing signs of lower respiratory tract involvement while maintaining an SpO_2_ level of 94% or higher in room air. Severe disease is characterized by tachypnea (rapid breathing), more than 50% lung involvement on imaging, an SpO_2_ less than 94% in room air, and a FiO_2_/PaO_2_ ratio of less than 300 mmHg.

### Data collection

CT scans were used to assess the type, pattern of spread, and severity of pulmonary involvement based on the severity score. The lungs were divided into five lobes, with severity scored as follows: Grade 1 for involvement less than 25%, Grade 2 for 25%–50%, Grade 3 for 50%–75%, and Grade 4 for involvement more significant than 75%. These individual scores were then summed to produce a total severity score. CT images were independently reviewed by two pediatric radiologists blinded to each other's interpretations. Common CT manifestations were analyzed about clinical symptoms, laboratory findings, disease severity, and outcomes**.**

### Statistical analysis

Descriptive statistics for qualitative data were reported as frequencies and percentages, while quantitative data were summarized using means, standard deviations, medians, and ranges. Statistical evaluations included the Kruskal–Wallis test, correlation coefficient analysis, and Pearson chi-squared test. Analyses were performed using SPSS software version 25.0, with a *p*-value of less than 0.05, which is considered statistically significant. Ethical principles for research were strictly adhered to throughout the study, ensuring the confidentiality of patient information.

## Results

### Study population

A total of 252 patients were enrolled in the study, comprising 147 boys (58.3%) and 105 girls (41.7%). The mean age of the patients was 71.2 months (±59.42), with an age range from 0.2 to 228 months. The demographic profile of patients is discussed in Table 1.

**Table 1:** Demographic characteristics of patients.

Demographic characteristics	Subgroup	Number (percentage)	Metric
Sex	Male	147 (58.3%)	
	Female	105 (41.7%)	
		Mean ± SD	Median (Range)
Age (Month)		71.2 ± 59.42	60 (0.2,228)

### Clinical outcomes

The most common clinical outcomes observed are shown in Table 2 and were as follows: fever was reported in 153 patients (67.4%), myalgia in 62 patients (27.3%), vomiting in 48 patients (21.1%), and diarrhea in 28 patients (12.3%). Abdominal pain was reported in the same number of cases as diarrhea. Other notable outcomes included poor feeding in 35 patients (15.4%), cough in 47 patients (20.7%), respiratory distress in 53 patients (23.5%), rash in 12 patients (5.3%), diabetic ketoacidosis (DKA) in 4 patients (1.8%), loss of consciousness (LOC) in 10 patients (4.4%), and seizures in 9 patients (4%). Fever was the most prevalent outcome, while DKA was the least common.

**Table 2:** Clinical outcomes.

Clinical outcomes	Frequency	Percentage%
Fever	153	67.4%
Myalgia	62	27.3%
Respiratory distress	53	23.5%
Vomiting	48	21.1%
Cough	47	20.7%
Poor feeding	35	15.4%
Abdominal pain	28	12.3%
Diarrhea	28	12.3%
Rash	12	5.3%
LOC	10	4.4%
Seizure	9	4.0%
DKA	4	1.8%

### Underlying health conditions

An analysis of underlying health conditions revealed that oncology-related diseases were the most frequently observed, affecting 34 patients (85%). In contrast, rheumatological conditions were the least common, affecting only 3 patients (1.3%). Other underlying conditions, ranked from most to least prevalent, included surgical issues in 26 patients (11.5%), neurological disorders in 19 patients (8.4%), gastrointestinal issues in 17 patients (7.5%), nephrotic syndrome in 16 patients (7%), cardiac conditions in 13 patients (5.7%), diabetes mellitus in 8 patients (3.5%), respiratory issues in 6 patients (2.6%), and metabolic disorders in 4 patients (1.8%). Overall, 121 patients (48%) had at least one underlying health condition, as shown in Table 3.

**Table 3:** Underlying health conditions.

Underlying Health conditions	Frequency	Percentage%
Oncology	34	85.0%
Surgical	26	11.5%
Neurology	19	8.4%
GI	17	7.5%
Nephrotic	16	7.0%
Cardiac	13	5.7%
DM	8	3.5%
Respiratory	6	2.6%
Metabolic	4	1.8%
Rheumatology	3	1.3%

### Severity of illness

Among the children, severe illness was noted, with 118 patients (52%) requiring ICU admission and 4 patients (1.8%) needing intubation. The agreement between ICU admission and intubation was assessed using the Kappa coefficient and Pearson correlation, indicating a very weak association between the two variables, as shown in Table 4. Ultimately, 204 patients (93.2%) were discharged, while 15 patients (6.8%) expired.

**Table 4:** ICU admission and need for intubation.

ICU admission or Intubation	Frequency	Percentage%	Kappa agreement	Pearson correlation
ICU	118	52.0%	0.033	0.129
INTUBATION	4	1.8%		

### Chest x-ray findings

Regarding chest x-ray (CXR) findings (Table 5), among the total data of 252 patients, 76 patients (30.2%) had abnormal CXR results, while 173 patients (68.7%) had normal CXRs, and 3 patients (1.2%) exhibited other findings. Among the 76 patients with abnormal CXRs, peribronchial patterns were the most prevalent, observed in 52 patients (68.4%), followed by ground-glass opacity in 15 patients (19.7%), and consolidation in 9 patients (11.9%).

**Table 5:** Chest x-ray findings.

CXR findings	Subgroup	Number (percentage)
CXR abnormalities		76 (30.2%)
	CXR PERIBROCHIAL	52 (68.4%)
	CXR CONSO	15 (19.7%)
	CXR GGO	9 (11.9%)
Normal		173 (68.7%)
Other		3 (1.2%)
Total		252 (100%)

### CT scan findings

CT scan findings were categorized based on type and location of involvement, detailed in Tables 6A,B. The most common type of involvement was mixed pattern, while cavitation was the least observed. Bilateral involvement was the most frequent location, whereas anterior dominance was the least common. Regarding pleural and pericardial effusions, unilateral pleural effusion was noted in 12 patients (7.9%) and bilateral effusion in 10 patients (6.6%), detailed in Table 7. The highest frequency of pleural effusion severity was mild, with 12 patients (7.9%) affected, while mild pericardial effusion was observed in 4 patients (2.7%). Investigating the associated findings on CT scans also showed that peri-bronchial cuffing was the most frequently observed finding, while LAP was the least common. In the end, we analyzed the findings from CT scans with the diagnostic imaging results for COVID-19. The most common diagnosis was “typical appearance,” observed in 56 cases (37.6%), while the least common was “indeterminate appearance,” noted in 17 cases (11.4%).

**Table 6:** (A,B) CT scan findings based on type and location of involvement.

(A) Type of Involvement	Frequency	Percentage%
Mixed pattern	32	21.10%
Non-lobar consolidation	31	20.50%
Ground glass opacity	30	19.70%
Round	16	10.50%
Lobar consolidation	15	9.90%
Linear	15	9.90%
Nodule	15	9.90%
Crazy paving	9	6.00%
Reverse halo	8	5.40%
Halo sign	5	3.30%
Cavitation	1	0.70%
(B) Location	Frequency	Percentage%
Bilateral	66	43.40%
Peripheral	63	41.40%
Multi Focal	57	37.50%
Posterior Dominancy	44	28.90%
Lower Dominancy	44	29.10%
Non Dominancy	28	18.40%
Unilateral	16	10.50%
Central	15	9.90%
Diffuse	13	8.60%
Unilateral Focal	10	6.60%
Upper Dominancy	7	4.60%
Anterior Dominancy	5	3.30%

**Table 7:** Pleural and pericardial involvement.

Pleural and pericardial involvement	Classification	Frequency	Percentage%
P/E Unilateral		12	7.9%
P/E Bilateral		10	6.6%
P/E Severity	.00	129	85.4%
	1.00	12	7.9%
	2.00	6	4.0%
	3.00	4	2.6%
Pericard Effusion	No	145	96.7%
	Mild	4	2.7%
	Moderate	1	0.7%
	Severe	0	0.0%

### CT scan scoring

CT scan scoring as shown in Table 8, revealed that out of the total 252 patients, 136 had CT scores recorded, with a highest frequency score of 2 seen in 16 patients (11.8%). The mean CT score was found to be 3 ± 4. In recent years, there has been growing interest in automated tools for disease severity assessment based on previously developed algorithms on chest CT, mainly within the context of COVID-19. Thoracic VCAR (Volume Computer-Assisted Reading) is one such tool that is an AI-based software that uses deep learning algorithms to quantify pulmonary involvement. It segments lung regions automatically, identifies abnormalities like ground-glass opacities (GGO), consolidations, and reticular patterns, and generates a severity score aggregating volumetric analysis. AI-based systems are more objective, have higher interobserver agreement and faster assessment time than manual scoring, which makes them attractive for high-throughput clinical settings.

**Table 8:** Ct scan scoring.

CT scan scoring	Subgroup of CT scores	Frequency	Percentage%
CT Score	0.00	65	47.8%
	1.00	9	6.6%
	2.00	16	11.8%
	3.00	6	4.4%
	4.00	15	11.0%
	5.00	7	5.1%
	6.00	4	2.9%
	7.00	2	1.5%
	8.00	3	2.2%
	10.00	1	0.7%
	11.00	1	0.7%
	12.00	1	0.7%
	13.00	1	0.7%
	14.00	1	0.7%
	15.00	1	0.7%
	16.00	1	0.7%
	17.00	2	1.5%
Mean/SD		3	4

Manual CT severity scoring showed a significant association with disease severity among pediatric COVID-19 patients in our study (*p* < 0.05). Mean CT severity score was 3 ± 4 (higher in children with ICU admission: 4 ± 5; mild disease: 2 ± 3). Our results align with previous studies that show a relationship between higher lung involvement on CT and worse clinical outcomes, such as intubation and mortality. In addition, certain CT abnormalities including non-lobar consolidation, mixed patterns, reverse halo sign, and multifocal or peripheral involvement were significantly associated with high disease severity (*p* < 0.05). These findings showcase the role of automated and AI-assisted applications like Thoracic VCAR in augmenting clinical decision-making through the standardized evaluation of severity and generating fast and reproducible assessments. Such AI-based systems integrated into clinical workflows may allow to assess the risk early on, decrease the workload of radiologists, and help improve patient outcomes, particularly in resources limited setups. Such technologies would need to be adapted for application in pediatric imaging, where verification of accuracy in different patient populations would be a must in future studies.

### Prognostic factors and CT scan findings

Prognostic factors for COVID-19 in children were analyzed concerning CT scan findings (Table 9). The presence of non-lobar consolidation, mixed pattern, reverse halo sign, multifocal involvement, bilateral involvement, peripheral involvement, posterior dominance, lower dominance, and bronchiectasis were all significantly associated with poor prognosis, indicated by the need for intubation, ICU admission, and mortality (all *P*-values <0.05).

**Table 9:** The correlation between the prognosis of COVID-19 in children and CT scan findings.

Prognosis	0	1	Total
Non lobar consolidation	Pearson chi2 (1) = 6.8318 Pr = 0.009		
0	62	8	70
1	57	23	80
Mixed pattern	Pearson chi2 (1) = 5.8193 Pr = 0.016		
0	62	9	71
1	57	23	80
Reverse halo	Pearson chi2 (1) = 7.3866 Pr = 0.007		
0	69	0	69
1	71	8	79
Multi focal	Pearson chi2 (1) = 10.8680 Pr = 0.001		
0	54	17	71
1	40	40	80
Bilateral	Pearson chi2 (1) = 6.9725 Pr = 0.008		
0	48	23	71
1	37	43	80
Peripheral	Pearson chi2 (1) = 6.3527 Pr = 0.012		
0	49	22	71
1	39	41	80
Posterior dominancy	Pearson chi2 (1) = 7.6113 Pr = 0.006		
0	58	13	71
1	49	31	80
Lower Dominancy	Pearson chi2 (1) = 3.9564 Pr = 0.047		
0	55	15	70
1	51	29	80
Bronchectasia	Pearson chi2 (1) = 3.7415 Pr = 0.053		
0	71	0	71
1	74	4	78

**Correlation Between Clinical Manifestations and CT Findings (Table 10)**

1. Patients with nodules significantly reported myalgia (*P*-value = 0.037).
2. Patients with round opacities had a significant incidence of diarrhea (*P*-value = 0.01).
3. Higher severity of pleural effusion was associated with increased abdominal pain (*P*-value = 0.001).
4. Patients with unilateral pleural effusion and non-lobar consolidation exhibited poor feeding symptoms.
5. Patients with mixed patterns and other specific findings exhibited increased cough frequency.
6. Dyspnea was significantly more prevalent among those with multifocal and peripheral involvement.
7. Rash was more commonly reported among patients with crazy paving patterns.
8. Loss of consciousness (LOC) was more prevalent among those with bilateral pleural effusion.
9. Seizures were notably higher in patients with bronchiectasis.

**Table 10:** The correlation between clinical manifestations and CT scan findings.

CT finding		Myalgia	Diarrhea	abd pain	POORFEEDING	Cough	Respiratoty distress	Rash	LOC	SEIZURE
CON LUBA	R	−0.122	0.107	−0.086	0.235	0.057	0.069	0.047	0.032	0.047
	P	0.172	0.233	0.337	0.008	0.526	0.439	0.598	0.719	0.598
MIX PAT	R	0.039	0.027	−0.093	−0.053	0.286	0.161	0.150	0.038	0.061
	P	0.663	0.763	0.299	0.556	0.001	0.071	0.092	0.671	0.498
ROUND	R	0.060	0.229	−0.103	−0.079	0.256	0.088	−0.081	−0.088	−0.081
	P	0.500	0.010	0.249	0.379	0.004	0.328	0.362	0.323	0.362
NODULE	R	0.186	0.017	0.045	−0.072	0.217	0.049	−0.079	−0.086	0.158
	P	0.037	0.851	0.615	0.422	0.014	0.584	0.379	0.340	0.077
CRAZY PAVING	R	0.070	−0.013	−0.128	−0.113	0.134	0.092	0.227	0.067	0.083
	P	0.433	0.883	0.155	0.207	0.134	0.306	0.010	0.454	0.357
MULTI FOCAL	R	0.030	−0.015	−0.071	−0.096	0.273	0.228	0.048	0.017	−0.028
	P	0.741	0.871	0.429	0.281	0.002	0.010	0.589	0.847	0.759
BILATERAL	R	0.099	0.045	−0.113	−0.088	0.290	0.164	0.101	−0.006	0.101
	P	0.268	0.614	0.205	0.325	0.001	0.066	0.257	0.946	0.257
PERIPHERAL	R	0.099	−0.033	−0.050	−0.069	0.281	0.266	0.037	0.006	0.037
	P	0.270	0.716	0.578	0.439	0.001	0.003	0.677	0.951	0.677
CENTRAL	R	0.045	0.107	−0.086	−0.063	0.179	0.012	0.047	0.032	0.047
	P	0.618	0.233	0.337	0.483	0.044	0.892	0.598	0.719	0.598
ANT DOMINANCY	R	0.261	0.067	0.037	−0.073	0.112	0.083	0.172	−0.044	−0.040
	P	0.003	0.452	0.683	0.413	0.210	0.355	0.053	0.627	0.654
POST DOMINANCY	R	0.027	0.011	−0.026	−0.019	0.203	0.149	0.098	0.144	−0.064
	P	0.764	0.902	0.768	0.832	0.022	0.095	0.275	0.107	0.472
UPPER DOM	R	0.128	0.012	−0.019	−0.098	0.191	0.073	−0.054	−0.058	−0.054
	P	0.150	0.891	0.829	0.272	0.032	0.415	0.548	0.515	0.548
LOWER DO	R	0.011	−0.030	−0.013	0.093	0.265	0.209	0.189	0.153	0.106
	P	0.905	0.738	0.883	0.299	0.003	0.019	0.034	0.086	0.237
NON DOMINANCY	R	−0.019	−0.055	−0.161	−0.132	0.124	0.193	−0.105	−0.114	−0.008
	P	0.836	0.537	0.070	0.138	0.166	0.029	0.241	0.204	0.926
P/E UNI	R	0.005	−0.013	0.116	0.239	−0.083	0.092	0.083	−0.067	−0.062
	P	0.958	0.883	0.196	0.007	0.357	0.306	0.357	0.454	0.490
P/E BILATERAL	R	−0.040	−0.098	0.138	0.073	0.008	0.184	0.095	0.221	−0.058
	P	0.652	0.271	0.121	0.415	0.925	0.038	0.288	0.012	0.519
COVID19 IMAGING	R	−0.105	0.023	0.073	0.030	−0.326	−0.258	−0.063	0.031	−0.007
	P	0.242	0.798	0.419	0.736	0.000	0.004	0.484	0.732	0.935
BRONCHECTASIA	R	−0.030	0.066	0.035	−0.075	0.223	0.081	−0.041	−0.044	0.172
	P	0.738	0.462	0.696	0.409	0.012	0.368	0.651	0.624	0.055
LAP	R	−0.089	−0.049	−0.059	−0.052	−0.072	−0.081	−0.029	0.246	0.270
	P	0.323	0.588	0.514	0.562	0.427	0.369	0.751	0.006	0.002
P/E SEVERITY	R	−0.061	−0.096	0.285	0.258	−0.081	0.192	0.036	0.127	−0.078
	P	0.497	0.282	0.001	0.003	0.367	0.030	0.690	0.156	0.385

Note: Correlation coefficients (R) indicate the strength and direction of the relationship between clinical manifestations and CT scan findings. A p-value of less than 0.05 is considered statistically significant, suggesting a meaningful association between the clinical symptom and the CT scan finding. Clinical manifestations include symptoms such as fever, myalgia, and respiratory distress, while CT scan findings include patterns like non-lobar consolidation, mixed patterns, and bilateral involvement.

### CT scan scoring and disease severity

There is a significant link between the CT score and disease severity (*p*-value=0.03). As shown in Table 11, The average disease score in patients with severe disease is 4 ± 5, while in those with mild disease, it is 2 ± 3.

**Table 11:** The correlation between disease severity and CT scan score.

Severity of disease with CT score	Total	Disease Severity			*P*-value
		Severe	Moderate	Mild	
CT SCORE	3 ± 4	4 ± 5	3 ± 4	2 ± 3	0.031
	(0,17)	(0,17)	(0,17)	(0,11)	

## Discussion

In the present study, we aimed to investigate the imaging patterns observed through CT scans in pediatric patients with COVID-19 in Iran. A total of 252 children were included, with an average age of 71.2 ± 59.42 months and a slight male predominance, as 58.3% of the participants were boys. This male-to-female ratio is consistent with findings from studies conducted in China (4) and the United States (3).

Fever emerged as the most prevalent symptom, reported in 67.4% of cases, followed by myalgia (27.3%), respiratory distress (23.5%), vomiting (21.1%), cough (20.7%), poor feeding (15.4%), and other gastrointestinal symptoms such as abdominal pain and diarrhea (12.3% each). Other symptoms were less common, each occurring in fewer than 10% of patients. These findings are comparable to previous research; for instance, Xia et al. (20) found fever (60%) and cough (65%) to be the most common symptoms among 13 evaluated children. Similarly, Du et al. (21) reported fever (43.4%) and dry cough (44.5%) among 182 patients, with gastrointestinal symptoms accounting for 11% of cases, including diarrhea, abdominal discomfort, and vomiting. A systematic review encompassing 43 articles and 158 confirmed pediatric COVID-19 cases also identified fever, cough, and vomiting as the most frequently reported symptoms (22). Notably, our study did not include any asymptomatic patients, as all participants were hospitalized due to symptomatic presentation.

In terms of clinical severity, 52% of the children required admission to the Intensive Care Unit (ICU), with 1.8% needing intubation. In comparison, a systematic review by Patel et al. (23) indicated that 26 out of 382 hospitalized children (6.8%) required ICU care, and among studies providing intubation data, 7 out of 327 hospitalized children (2.1%) with COVID-19 required this intervention. The high rate of ICU admissions in our study may be attributed to the underlying health conditions of our patients, as well as the impact of COVID-related social restrictions, which limited access to medical centers to only those with severe symptoms. The observed mortality rate in our study was 7.3%. In contrast, Patel et al. (23) reported a mortality rate of approximately 0.18%. The higher mortality rate in our study may be attributed to the fact that we focused specifically on hospitalized patients, whereas the systematic review included all children diagnosed with COVID-19. Consistent with our findings, the CDC has noted that while children generally experience milder symptoms, severe cases do occur within this population, and fatalities have been documented (3).

In our study, we found that the most common underlying condition among participants was oncology-related diseases, affecting 34 children (85%). This was followed by 26 children (11.5%) who had undergone surgical procedures, while rheumatological diseases were the least prevalent, affecting only 3 children (1.3%). These findings are consistent with a large multicenter retrospective study conducted in the United States, which indicated that pediatric patients with cancer face a greater risk of severe COVID-19 infection compared to their peers in the general pediatric population (24).

In our study, 152 children underwent CT scans, revealing that 43.4% exhibited bilateral lung involvement, while GGOs were observed in 19.7% of the cases. The halo sign appeared in 3.3% of patients, and a combination of lobar and non-lobar consolidation was present in 30% of the participants. Unilateral pleural effusion was noted in 12 children (7.9%), and 10 children (6.6%) had bilateral pleural effusion. Additionally, mild pericardial effusion was detected in four patients (2.7%). It is hypothesized that due to the generally milder manifestations of COVID-19 in children, the associated chest imaging findings tend to be less pronounced and may even appear normal in some cases. For instance, a study involving 30 pediatric patients found that all nine asymptomatic individuals had normal chest CT results (25). In a systematic review and meta-analysis conducted by Nino et al. (17) it was reported that 27.7% exhibited bilateral lung lesions. The predominant CT findings among pediatric patients with COVID-19 was similar to our study, and included GGOs (37.2%) and consolidations or pneumonic infiltrations (22.3%). Notably, common imaging features associated with viral respiratory infections in children, such as perihilar symptoms and hyperinflation, were absent in those diagnosed with COVID-19. Pleural effusion or adjacent pleural thickening was detected in only 0.5% of cases, with no instances of pericardial effusion reported. Similar to the previous study, perihilar involvement and hyperinflation were not observed in our participants.

Similarly, Ghodsi et al. (26), also found GGOs as the predominant chest CT finding, observed in 71.43% of cases. This was followed by peribronchial thickening (60%), linear or band opacities (32.8%), consolidation (28.57%), nodules (18.57%), effusion (7.14%), and focal lucency (7.14%). Consistent with previous data, the majority of cases presented with bilateral abnormalities. The authors highlighted distinct patterns of pulmonary involvement compared to other studies focused on the pediatric population, which may be attributed to the age factor, as their study exclusively examined children under one year of age.

Additionally, a review of thirty-nine observational studies examining chest CT scans in children with COVID-19 revealed abnormal findings in 717 out of 1,028 subjects (27). This review noted a higher prevalence of lower lobe involvement and unilateral lung lesions, contrasting with our findings and those of Nino et al. While the authors did not disclose the mean age of their participants, Yoon et al. (22) reported that typical GGOs were more frequently observed in older children (83.3%) compared to only 20.8% in younger children. This suggests that age variations within the pediatric population may influence the typical presentations seen on CT scans. Overall, these findings align with the lung abnormalities documented in adult COVID-19 patients (28); however, they were observed to be less frequent and less severe in children.

Several factors may account for the observed age-related differences in lung findings associated with COVID-19 (17). Variations in the pathobiology of COVID-19 between adults and children could lead to distinct disease patterns. For example, children with SARS-CoV-2 infection are significantly less likely to experience involvement of the pleural space. Also, older adults with COVID-19 often have pre-existing chronic lung conditions that are typically absent in previously healthy children infected with SARS-CoV-2.

Chest x-ray (CXR) abnormalities were observed in only 39.7% of cases, while the remaining images appeared normal. The most frequently noted abnormality was peribronchial involvement, accounting for 25.2%, followed by GGOs at 19.7% and consolidation at 11.9%. Previous studies have identified common CXR findings such as opacities, pneumonic infiltrates, and lung haziness, often referred to as “white lung” (29–31). These radiographic features may represent the GGOs associated with SARS-CoV-2 infection.

Our analysis indicates that abnormalities identified in CT scans are correlated with a poor prognosis, including increased likelihood of intubation, ICU admission, and mortality. These findings suggest that the presence of certain abnormalities on CT scans can serve as a marker for worse disease outcomes. Furthermore, CT scan results are closely linked to the severity of the clinical condition. Notably, children exhibiting higher CT scores tend to present with more severe cases of the disease, highlighting the need for increased monitoring and potential hospitalization in ICU settings for these patients. Our results align with those of Ali et al. (18), who found that abnormal radiological findings were significantly more prevalent among severe and critical cases, and that radiological severity scores correlated strongly with symptom severity. This is consistent with prior studies on COVID-19, which demonstrated that patients classified as severe often had elevated CT involvement scores (32). Li et al. (33) and Sun et al. (30) also reported that high CT scores were characteristic of children with severe or critical COVID-19 conditions. In our study, the average CT scan score for patients in severe condition was 4 ± 5, compared to 2 ± 3 for those with mild disease, which is similar to the mean CT score of 6.5 reported for severe cases by Ali et al. (18). Moreover, we explored the relationship between specific CT scan presentations and clinical symptoms. For instance, nodules were associated with muscle pain, completely round lesions with clear boundaries correlated with diarrhea, severe pleural effusions were linked to abdominal pain, unilateral pleural effusions coincided with poor feeding, and bilateral pleural effusions and lymphadenopathy were associated with decreased levels of consciousness. Bronchiectasis and lymphadenopathy were related to seizures. Additionally, we found that most complications observed on CT scans were tied to pulmonary symptoms such as cough and shortness of breath. In line with this, Du et al. (21) reported that patients with pneumonia exhibited a higher incidence of fever and cough. To further elucidate the relationship between CT scan abnormalities and non-respiratory symptoms, additional research is necessary.

Several limitations must be acknowledged in this study. First, the cross-sectional design did not allow for differentiation of CT scan findings based on the disease's progression. Previous research indicates that while baseline CT scans may appear normal, subsequent imaging can reveal disease-related lung involvement (34). Second, our analysis did not include diagnostic measures for COVID-19, such as PCR results, which could have provided critical insights into the relationship between serology testing and imaging, as well as the sensitivity of CT scans as a diagnostic tool. Lastly, considering the potential impact of age on imaging patterns, categorizing patients by age group and examining the imaging findings within each group may have yielded additional valuable insights.

### Limitation

There are several limitations of this study. First, its retrospective nature may lead to selection bias, favoring only inpatients in whom CT scans are available. Second, disease progression was not controlled, as CT scans were performed at different points of illness. Lastly, this is a single-center study and may limit its generalizability to larger pediatric populations. Prospective designs and multicenter validation, as well as AI-assisted imaging analysis, should be the focus of future research to enhance severity assessment and clinical decision-making.

## Conclusion

We found that CT severity scores significantly correlated with clinical outcomes, including ICU admissions, intubation rate, and death (*p* = 0.031). Of importance, mean CT severity scores were higher among patients with severe disease (4 ± 5) compared to patients with mild disease (2 ± 3). Moreover, certain CT findings, including the presence of non-lobar consolidation, mixed patterns, reverse halo sign, multifocal involvement and peripheral distribution of the lesions, are significantly associated with worse prognosis (*p* < 0.05). These imaging findings can be used as prognostic factors for assessing patients who are at risk and may eventually need intensive care treatment.

Automated CT scoring tools based on AI (e.g., the software named Thoracic VCAR), could improve the accuracy and performance of severity assessment. Objective, reproducible, and rapid quantification of lung involvement using these pneumonitis and ILD systems could help to standardize disease classification, enable early risk stratification, and potentially optimize treatment strategies—even in resource-limited settings. The next step would be to validate cutting-edge strategies such as AI-assisted severity scoring on the pediatric population, which can help to inform therapeutic and diagnostic decisions. In conclusion, augmenting imaging analytics with clinical evaluations can greatly optimize diagnosis, management and outcomes of children suffering from COVID-19 and other pulmonary diseases.

## Data Availability

The original contributions presented in the study are included in the article/Supplementary Material, further inquiries can be directed to the corresponding authors.
